# Nondestructive 3D Image Analysis Pipeline to Extract Rice Grain Traits Using X-Ray Computed Tomography

**DOI:** 10.34133/2020/3414926

**Published:** 2020-05-02

**Authors:** Weijuan Hu, Can Zhang, Yuqiang Jiang, Chenglong Huang, Qian Liu, Lizhong Xiong, Wanneng Yang, Fan Chen

**Affiliations:** ^1^Crop Phenomics Joint Research Center, Wuhan 430070, China; ^2^Institute of Genetics and Developmental Biology Chinese Academy of Sciences, Beijing 100101, China; ^3^Britton Chance Center for Biomedical Photonics, Wuhan National Laboratory for Optoelectronics, and Key Laboratory of Ministry of Education for Biomedical Photonics, Department of Biomedical Engineering, Huazhong University of Science and Technology, Wuhan 430074, China; ^4^National Key Laboratory of Crop Genetic Improvement, National Center of Plant Gene Research, Agricultural Bioinformatics Key Laboratory of Hubei Province, and College of Engineering, Huazhong Agricultural University, Wuhan 430070, China

## Abstract

The traits of rice panicles play important roles in yield assessment, variety classification, rice breeding, and cultivation management. Most traditional grain phenotyping methods require threshing and thus are time-consuming and labor-intensive; moreover, these methods cannot obtain 3D grain traits. In this work, based on X-ray computed tomography, we proposed an image analysis method to extract twenty-two 3D grain traits. After 104 samples were tested, the *R*^2^ values between the extracted and manual measurements of the grain number and grain length were 0.980 and 0.960, respectively. We also found a high correlation between the total grain volume and weight. In addition, the extracted 3D grain traits were used to classify the rice varieties, and the support vector machine classifier had a higher recognition accuracy than the stepwise discriminant analysis and random forest classifiers. In conclusion, we developed a 3D image analysis pipeline to extract rice grain traits using X-ray computed tomography that can provide more 3D grain information and could benefit future research on rice functional genomics and rice breeding.

## 1. Introduction

Rice is one of the most important food crops worldwide, especially in China [1–3]. There is an urgent need to produce high-yield and resistant rice to cope with population growth, climate change, and increased numbers of pests and diseases [4–6]. Phenotypes have been shown to greatly accelerate the process of rice genetics and breeding [7, 8]. The rice panicle, an important agronomic component [9], is closely associated with yield. In particular, the number of grains per panicle directly determines rice yield [10]. Thus, accurately quantifying the grain number and grain size per panicle is a key step in rice phenotyping [11]. Traditionally, the phenotyping of grain traits is manually performed after threshing; unfortunately, this is an incredibly time-consuming and labor-intensive process [12].

An image-based analysis is widely used in the measurement of grain traits. Yang developed a machine vision-based, integrated, and labor-free facility for automatically threshing panicles and evaluating rice yield-related traits [13]. Liu et al. combined X-ray digital radiography with a CCD camera to distinguish between filled and unfilled rice spikelets [14]. Whan et al. reported a fast, low-cost method for grain size and color measurements using a scanner [15]. These methods require manual threshing and thus are also time-consuming. Recently, some researchers have proposed techniques known as panicle measurement methods. Gong et al. proposed using the projected area and contour of a grain to build a prior edge wavelet correction model to approximately count the number of grains per panicle [16]. Zhao et al. established a method integrating image analysis with a 5-point calibration model to achieve the fast estimation of the number of spikelets per panicle [17]. Wu et al. reported a method that was used to recognize and quantify the number of grains per panicle using deep learning [18]. Adam et al. designed a panicle trait phenotyping tool [19] that can be used to measure a variety of traits, including the panicle length, the number of branches, the order of branches, the number of grains, and the grain size. Jhala et al. proposed a method to study rice panicle development through the projection of images of rice panicles taken by X-ray computed tomography [20]. These methods require the sample to be manually spread out before imaging and thus suffer from various shortcomings; for example, the measurement accuracy depends on manually preprocessing the panicle, which is time-consuming. In addition, these methods are capable of extracting 2D traits but lack the ability to measure 3D grain traits, such as grain volume and grain thickness.

Some 3D nondestructive measurement methods, such as X-ray microcomputed tomography (CT) and magnetic resonance imaging (MRI), could be used to noninvasively obtain the internal structure information of a sample [21–24]. Karunakaran et al. identified wheat grains damaged by the red flour beetle using X-ray images [25]. Recently, a method based on X-ray computed tomography has been successfully applied to the measurement of 3D grain traits. Strange automatically estimated the morphometry of a wheat grain from computed tomography [26]. Xiong et al. developed a 3D morphological method to complete the processing of computed tomography images of wheat spikes [27]. Hughes et al. achieved the nondestructive and high-content analysis of wheat grain traits using X-ray microcomputed tomography [28]. Le et al. further analyzed the morphological structural characteristics of wheat grains by microcomputed tomography [29]. As seen from the above, methods based on X-ray computed tomography are effective and nondestructive approaches for analyzing grain traits. To the best of our knowledge, few studies have been performed on measuring the 3D traits of rice grains. Su and Chen proposed a method for measuring the traits of rice panicles based on 3D microfocus X-ray computed tomography [30] but extracted only the number of spikelets. Nevertheless, rice grain traits, including the grain size, volume, and surface area, are of great significance for research on rice genetics and rice breeding. Therefore, a new method is needed to measure the 3D traits of rice grains.

This study proposed a high-throughput 3D image processing pipeline for extracting 22 grain traits based on X-ray CT. In addition, the relationships between the extracted traits and grain weight were studied, and these traits were used to classify rice varieties.

## 2. Materials and Methods

### 2.1. Experimental Materials and Image Acquisition

In this study, during the summer of 2019, one wild type (ZH11) and eight mutants produced by EMS mutagenesis were grown in Hainan Province. After their harvest, 24 panicles of the wild type and 10 panicles of each mutant, reaching a total of 104 panicles, were randomly selected for further analysis. The X-ray CT scanning system used in this study was developed by the Institute of Genetics and Developmental Biology, Chinese Academy of Sciences (IGDB, CAS, Beijing, China). The voltage and current were set as 90 kV and 3.2 mA, respectively. The panicle was placed in a plastic holder during the scan. The system was operated in fast and continuous scan mode, and 450 projection images were collected over a 360° rotation of each sample in 0.8° steps, which generated a 3D volume with a resolution of ~0.3 mm (512 × 512 × 450voxels, unsigned 16 bits integers). The CT reconstruction is achieved by Shennong-CT V1.0 (IGDB, CAS, Beijing, China). The total scanning and reconstruction time was ~2 minutes for each panicle. After the CT scanning was complete, all the samples were threshed manually, dehulled, and finally measured by a yield traits scorer (YTS) [13] to extract grain number, grain length, and grain weight.

### 2.2. Image Processing and Analysis Pipeline for Extracting Rice Grain Traits

**Figure 1:**
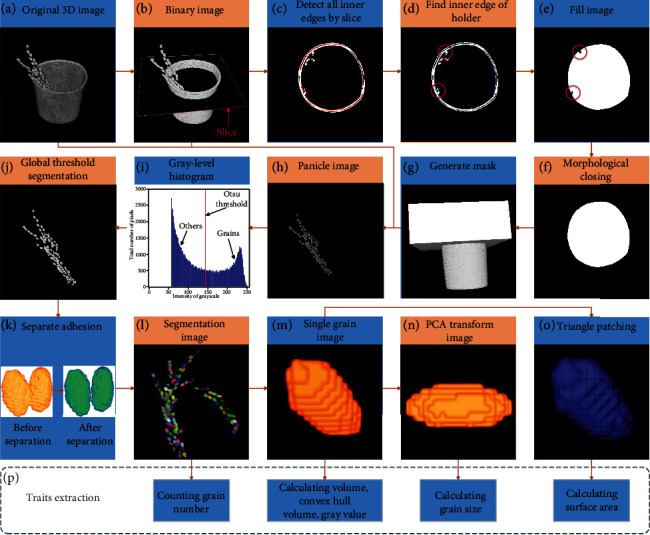
3D image processing pipeline based on X-ray CT and trait extraction. (a) Original CT image. (b) Binary image obtained using global adaptive threshold segmentation based on Otsu. (c) Detection of inner edges slice by slice. (d) Finding the inner edge of the holder by an appropriate size threshold. (e) Filling to get binary image. (f) Morphological closing operation. (g) Mask generation. (h) Obtaining a grayscale image of the panicle obtained by steps a, b, and g. (i) Grayscale histogram. (j) Grain segmentation by the global adaptive segmentation method based on Otsu. (k) Separation of overlapping grains by the improved watershed algorithm based on the Euclidean distance transform. (l) Segmented image. (m) Single grain image. (n) Grain image after PCA transform. (o) Triangle patching on the grain surface. (p) Trait extraction.

We developed a robust pipeline for automatically processing CT images and extracting rice grain traits. A flow chart of the main algorithm used to extract rice grain traits, including the preprocessing of images and extraction of traits, is shown in Figure 1. The workflow is described in detail as follows: (1) After image reconstruction, the CT images are saved slice by slice along the *z*-direction in DICOM format (Figure 1(a)). (2) The holder is removed (Figures 1(b)–1(h)). (3) 3D segmentation of rice gains is performed (Figures 1(h)–1(k)). (4) The number of grains is counted (Figure 1(l)). (5) Individual grain traits are calculated (Figure 1(m)). (6) The grain size is calculated using the principal component analysis (PCA) transform (Figure 1(n)). (7) The grain surface area is calculated by surface reconstruction (Figure 1(o)). All image processing and trait extraction procedures are performed through MATLAB 2018a, and the source codes of all the scripts are available online in Supplementary File 3 or at the following link: http://plantphenomics.hzau.edu.cn/download_checkiflogin_en.action, or https://github.com/cancanzc/ricePanicle_grainTraits_Processing. The usage instructions of the 3D image analysis pipeline are available in Supplementary File 2 and demonstrated in Supplementary Video 1.

### 2.3. Holder Clearance

**Figure 2:**
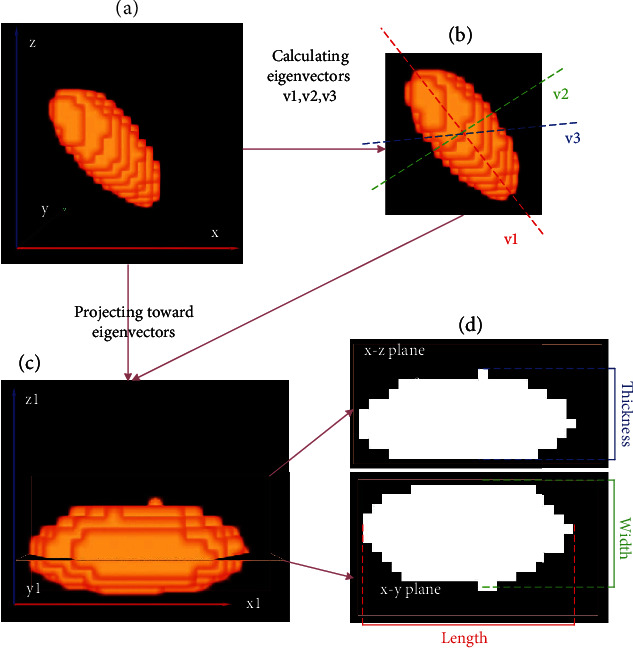
Calculating grain size by the PCA transform.

**Figure 3:**
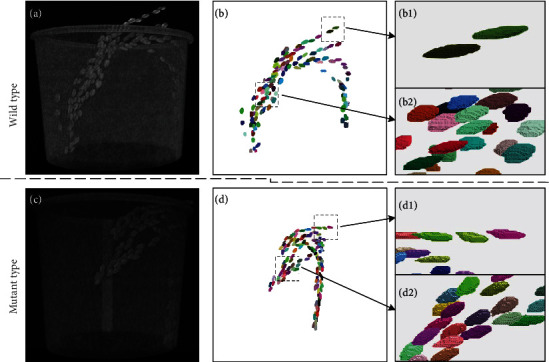
3D visualization of grain segmentation results. (a-c) Original 3D CT images of wild type and mutant rice panicles. (b-d) Segmentation results of the wild type (a) and mutants (c), respectively. (b1, b2) The details in the dashed box in b. (d1, d2) The details in the dashed box in d.

**Figure 4:**
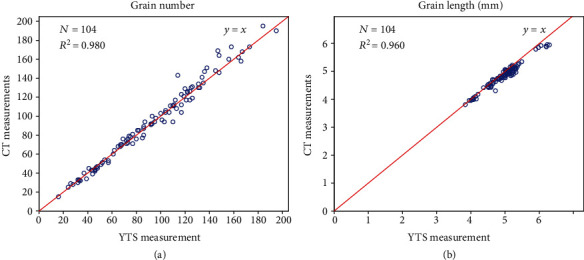
Comparison between the CT and YTS measurements. Performance evaluation for the grain number and grain length. Scatter plots of the CT measurements versus the YTS measurements for the grain number (a) and grain length (b).

**Figure 5:**
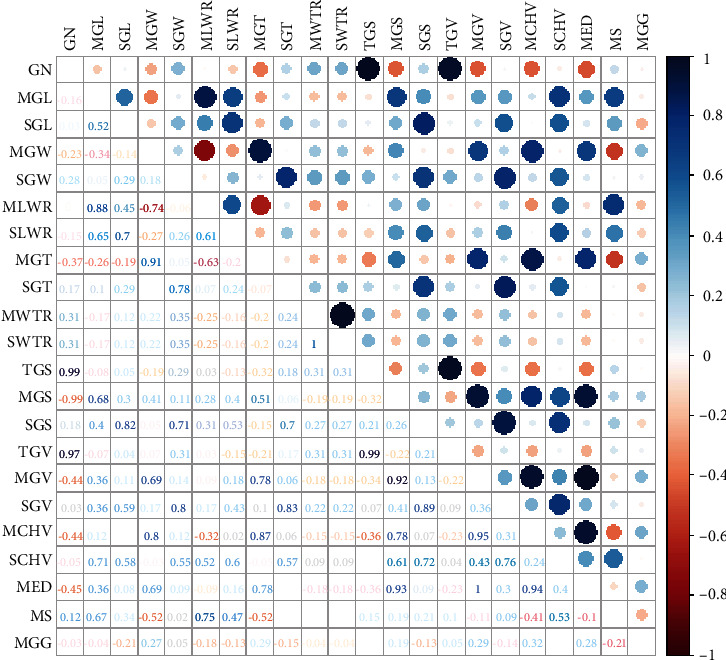
Correlation analysis between the 22 phenotypic traits.

The original CT data were stored slice by slice in DICOM format, and each sample was 226 megabytes in size. Each rice panicle was fixed in a holder when scanned. The holder must be removed for further processing of the 3D image. However, the rice panicle and the inner edge of the holder were close to each other during the scanning process and exhibited attenuation characteristics similar to X-ray absorption, which led to the complete overlap of their gray-level histograms; hence, it was difficult to remove the holder from the 3D image using a fixed grayscale threshold. Here, the following method of finding the holder edge was used to solve this problem: (1) first, a binary image was acquired (Figure 1(b)); (2) all the inner edges were detected for each slice along the *z*-axis (Figure 1(c)), and the appropriate threshold was selected to obtain the inner edge of the holder (Figure 1(d)); (3) the interior of the holder region was filled (Figure 1(e)); (4) a morphological closing operation was performed (Figure 1(f)); (5) the complete holder was obtained to generate a mask (Figure 1(g)); and (6) the mask was applied to the original image (Figure 1(a)) to remove the holder (Figure 1(h)).

### 2.4. 3D Image Processing

After removing the holder, grains, branches, impurities, and background remain in the 3D image (Figure 1(h)). Their combined gray-level histogram, which is bimodal in nature, is shown in Figure 1(i). The brighter peaks are grains, while the darker peaks are the background. The maximum variance between classes (Otsu) is an adaptive threshold determination method, which is mainly suitable for the segmentation of images with a large difference between foreground and background. Thus, the global adaptive segmentation method based on Otsu [31] was used to segment the grains (Figure 1(j)). The Otsu threshold (Figure 1(i)) was calculated by the Otsu method.

There is a trade-off between the image resolution and field of view. Therefore, ensuring a large imaging field of view at the expense of a low image resolution enabled us to scan large samples, which also increased the possibility for objects to be connected. To process connected grains, a separation algorithm was developed based on a watershed method applied to the distance transform of the binary image [32]. The watershed algorithm has been suggested to be an effective image region segmentation method, and its main use is to regions based on the gradient of the gray level image [33, 34]. The watershed algorithm used in this article was improved, and the detailed steps are as follows: (1) For each white pixel in a 3D image, the distance to the nearest black pixel was computed using a chessboard method for distance measurements. (2) Local maximum regions were detected by selecting an appropriate threshold related to the radius of the object. The regions with a distance less than threshold should belong to the same object, and these regions should be merged together. (3) With this improved computed distance map, the standard watershed algorithm was applied to find dividing contour lines (Figure 1(k)).

### 2.5. Grain Size Extraction

PCA [35] was applied to calculate the grain size. The principle of PCA is to transform data from the original three-dimensional space to another three-dimensional space by an orthogonal transform, the directions of which are determined by the three directions with the largest variance of data. The PCA method is described in detail as follows: (1) the grain is defined in the original coordinate space (*x*-*y*-*z*) (Figure 2(a)); (2) the 3D coordinates of the centers of all voxels of the grain are extracted as input features, and the eigenvectors (v1, v2, and v3) are calculated (Figure 2(b)); (3) Projecting the grain toward the eigenvectors, and the grain is transformed into a new coordinate space (*x*1-*y*1-*z*1) (Figure 2(c)); and (4) the grain length, width, and thickness are calculated by computing min and max coordinates of transformed voxels along each dimension, as shown in Figure 2(d).

### 2.6. Grain Volume, Surface Area, and Grain Number Count

After grain segmentation, the connected components were found. Then, the grain volume was calculated simply by counting the number of all voxels in each connected domain. The surface of each grain was jagged and not smooth. Thus, the surface of the grain was first reconstructed by using marching cubes [36, 37], then its surface area is computed by summing area of individual triangles. The total number of grains could also be directly counted. The definitions and abbreviations of the 22 phenotypic traits (grain number, grain size, 3D grain architectures, and grain density) are shown in Table 1. All of the 3D image analysis pipelines was developed using the MATLAB 2018a software (MathWorks, USA).

### 2.7. Stepwise Discriminant Analysis Statistical Method

Stepwise discriminant analysis (SDA) is an effective classification method [38] that involves the selection of features and the establishment of discriminant functions. For feature selection, the specific steps are as follows: All traits are selected as the input variables of the algorithm. There are no variables in the model at the beginning. First, the SDA algorithm will select the variable with the largest discriminant ability. Then, the second variable will be required to have the largest discriminant ability among the remaining variables. Because of the interrelationship among the different variables, the previously selected variable may lose its significant discrimination ability after selecting a new variable. Thus, every time a new variable is chosen, we must inspect the discrimination abilities of all previously selected variables to find all disabled variables and remove them. With this process, we find new variables until there are no variables that meet the requirements. In this study, stepwise discriminant analysis was performed by using the SPSS v.24 software (SPSS, USA), and the conditions for the probability of entry and deletion were set to 0.05 and 0.1, respectively.

### 2.8. Support Vector Machine Statistical Method

The support vector machine (SVM) is a pattern recognition method based on the principle of minimum structural risk [39]. The main idea is to map the data in the sample space to a higher-dimensional space and find a hyperplane in the higher-dimensional space so that the distances between the hyperplane and the different sample sets are as large as possible to ensure the minimum classification error rate. The radial basis function (RBF) is chosen as the kernel function of the support vector machine. The selection of the penalty coefficient *c* and kernel function parameter *γ* will affect the recognition ability of the support vector machine algorithm. Particle swarm optimization (PSO) is a swarm intelligence optimization algorithm [40] that can quickly find the optimal values of *c* and *γ* in a large range to improve the search efficiency and recognition accuracy of the algorithm. In this study, the implementation of the SVM algorithm is based on the MATLAB libsvm-3.23 toolbox.

### 2.9. Random Forest Statistical Method

Decision trees are a common class of machine learning algorithms based on the structure of a tree for making decisions [41]. A random forest (RF) consists of multiple decision trees, and there is no correlation between each pair of decision trees. Random forests employ the random selection of attributes in the training process of the decision tree. For each node of the decision tree, a subset of *k* attributes is randomly selected from the node attribute set, and then an optimal attribute is selected from the subset for division. Each sample subset is used to train a decision tree as a classifier. For an input sample, each tree will have a classification result, and the random forest will specify the category with the highest number of votes as the final classification result. In this study, the open-source random forest matlab toolkit [42] was used to build the random forest classifier.

## 3. Results and Discussion

### 3.1. Grain Segmentation and Phenotypic Trait Extraction

We developed a robust pipeline for automatically processing CT images and extracting 22 rice grain traits. The segmentation examples for the wild type and mutants are shown in Figure 3. The results of the pseudocolor image demonstrate that the grains were well recognized. After image segmentation, we extracted 22 phenotypic traits, including the grain number, grain shape, and grain size. The definitions and abbreviations of the phenotypic traits are shown in Table 1. An example of the segmentation result is shown in Supplementary Video 2.

### 3.2. Performance Evaluation of the Grain Trait Extraction

In the experiment, a total of 104 panicles were measured both automatically using X-ray CT and using a yield traits scorer (YTS) [13]. Regression analysis was applied to evaluate the measurement accuracy. The *R*^2^ values (formula (1)) between the CT and YTS measurements for the grain number and grain length were 0.980 and 0.960, respectively (Figure 4(a)–4(b)), and the root mean square error (RMSE, formula (2)) values of the CT measurements versus the YTS measurements for these two traits were 6.2 and 0.15 mm, respectively. The mean absolute percentage error (MAPE, formula (3)) of the automatic versus YTS measurements for the grain number and grain length was 4.65% and 2.41%, respectively. All the phenotypic data are shown in Supplementary File 1.

(1) $$\begin{array}{c} R^{2}=1-\frac{\sum_{\text{i}}{\left(x_{i}-y_{i}\right)}^{2}}{\sum_{\text{i}}{\left(x_{i}-\bar{y}\right)}^{2}} \end{array}$$

(2) $$\begin{array}{c} RMSE=\sqrt{\frac{\sum_{i}{\left(x_{i}-y_{i}\right)}^{2}}{n}} \end{array}$$

(3) $$\begin{array}{c} MAPE=\frac{1}{n}\sum_{i}\frac{\left|x_{i}-y_{i}\right|}{x_{i}}\times 100\% \end{array}$$

Where *n* is the total number of measurements, x_i_ is YTS measurements, y_i_ is CT measurements, and $\bar{y}$ is the mean value of the CT measurements.

### 3.3. Correlation Analysis between the 3D Grain Traits and Grain Weight

After a total of 22 traits were extracted from the 104 panicles, we explored the correlations between these traits. The correlation matrix among all the traits is shown in Figure 5. It can be seen from this figure that the total volume has strong correlations with the total surface area and the number of grains. In addition, the individual grain volume has relatively high correlations with grain surface area, grain width, and grain thickness and a relatively low correlation with grain length, which indicates that grain volume is more affected by grain width and grain thickness than by grain length. The correlations between the surface area and the length, width, and thickness of individual grains are similar, which indicates that grain length, width, and thickness have the same effect on the surface area of a grain.

**Figure 6:**
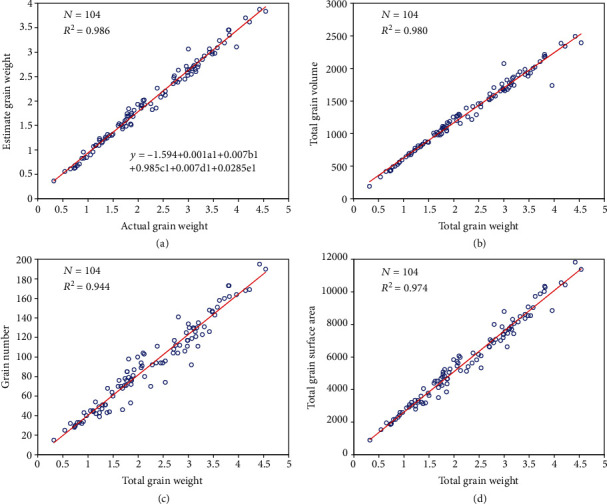
Relationship between the 3D grain traits and grain weight. (a) Scatter plots showing the relationship between the actual grain weight and the estimated grain weight using the 5 traits (a1, b1, c1, d1, and e1 represent TGV, GN, SGW, MGG, and SGL, respectively, (see Table 1 for their definitions)). Scatter plot of grain weight versus total grain volume (b), grain number (c), and total grain surface area (d).

Furthermore, to study the relationships between all the traits and the total grain weight, we performed stepwise discriminant analysis (SDA). The first feature selected is total grain volume (TGV), and the remaining selected features include GN, SGW, MGG, and SGL (see Table 1 for their definitions). When the five selected features were used, up to 98.6% of the variance in grain weight could be explained (Figure 6(a)). In addition, the *R*^2^ values between the total volume, grain number, and total surface area and the total grain weight were all above 0.94 Figures 6(b)–6(d).

### 3.4. Variety Classification Results

**Figure 7:**
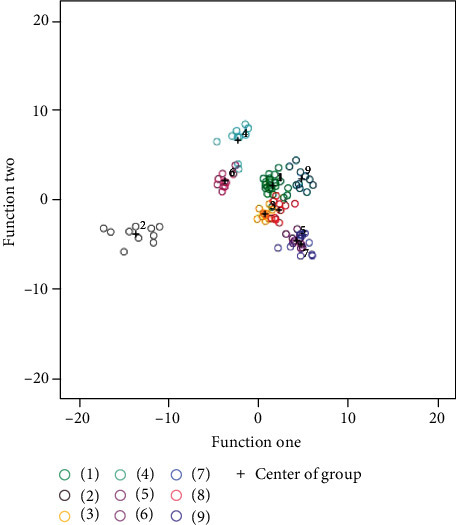
Classification results of different grain varieties using SDA. Stepwise discriminant analysis classification results. The abscissa and ordinate represent two classification functions that were built by a stepwise discriminant analysis algorithm. The black plus signs reflect the centers of different groups, which can be calculated with two decision functions. Dots of different colors represent different varieties.

In the experiment, we established three different models, namely, SDA, SVM, and RF models, to classify the rice varieties. Using stepwise discriminant analysis, a total of 13 traits (MLWR, GN, MGV, MGW, MGL, SWTR, MED, TGS, SGV, MGS, TGV, MGT, SGW, see Table 1 for their definitions) were selected for further classification analysis. Using two discriminant functions generated by the SDA algorithm, the grain variety classification results are visualized in Figure 7, which shows that most varieties were well classified.

To provide a convincing result in the case of limited samples, we used leave-one-out cross-validation (LOO-CV) to evaluate the established model. Leave-one-out cross-validation means that the sample was divided into 104 groups. For each test, one group was taken as the test set, while the rest were used as the training set. A total of 104 tests were performed, and their average value was used as the final classification accuracy of the model. The average recognition accuracies of the leave-one-out cross-validation method for the SDA, SVM, and RF models were 92.3%, 94.2%, and 92.3%, respectively. A comparison of the cross-validation results using the three models is shown in Table 2. We found that the SVM model was the best among the three models with 94.2% recognition accuracy.

### 3.5. Overlapping Grain Segmentation Based on the Improved Distance Transform Watershed Algorithm

**Figure 8:**
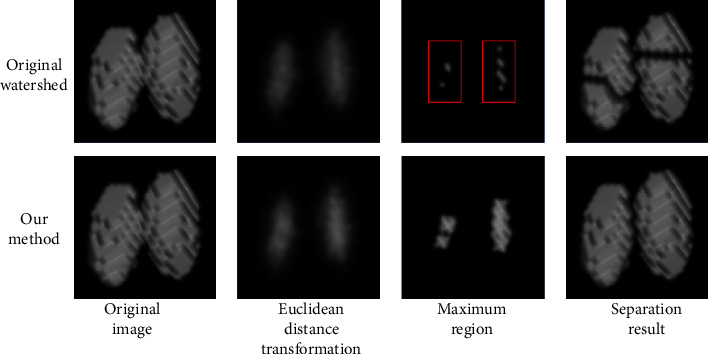
Overlapping grain segmentation. The top images represent the results of directly using traditional watershed segmentation based on a distance transform. The bottom images are the segmentation results using the improved method.

After segmentation, there is still some overlap between the grains. The improved watershed algorithm was used to separate overlapping grains (Figure 8). The top images represent the results of directly using the common watershed segmentation algorithm based on the distance transform, while the bottom images reflect the segmentation results using the improved method. The red rectangle in the figure indicates that directly using the traditional watershed algorithm will lead to the detection of multiple maxima within the grain, so separation will ultimately occur inside the grain. In contrast, our method separates grains only where they overlap.

### 3.6. Segmentation Results of Whole Rice Panicles

**Figure 9:**
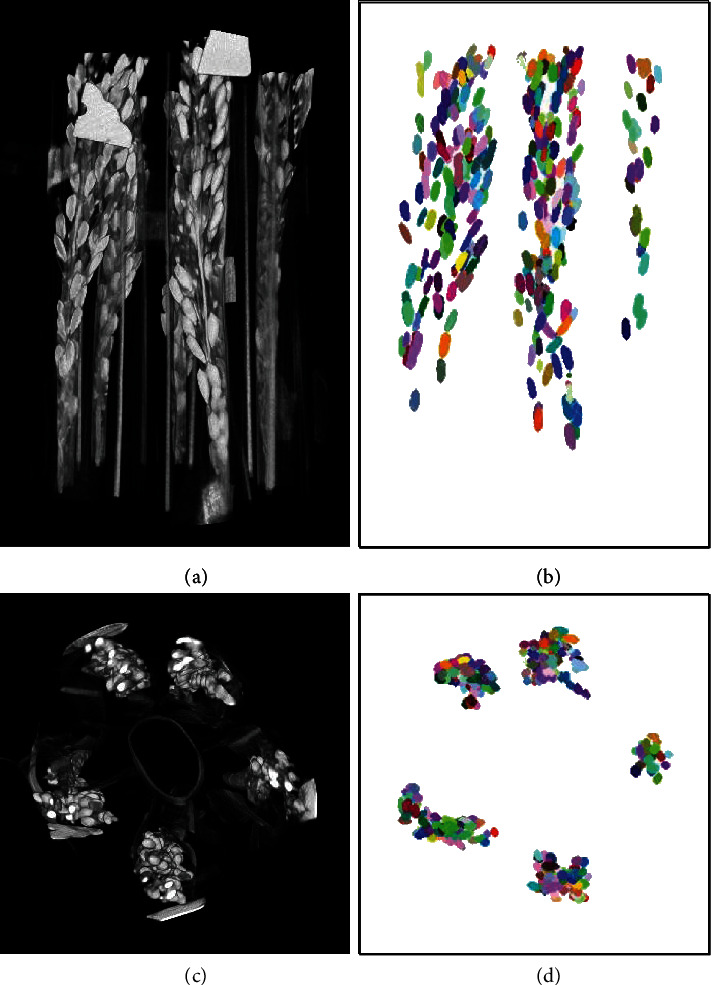
Visualization of the segmentation results of all panicles for a whole rice plant. Results of segmenting all the rice panicles in a rice plant by the proposed method. (a, c) Original images of the rice panicles from the side and top, respectively, and (b, d) the corresponding segmentation results.

To demonstrate the robustness and broad applicability of our method, we also implemented the segmentation of all rice panicles in a rice plant using X-ray CT (Figure 9 and Supplementary Video 3). To ensure that all the rice panicles were in the CT field of view, the panicles were wrapped by using a thin roll of paper when scanning. The segmentation results show that our method can accurately segment all the panicles of a rice plant and could be used to monitor the 3D dynamic development of panicles in the future.

### 3.7. The Comparison with Other 3D CT Image Analysis Pipelines to Extract Grain Traits

At present, most of the existing 3D CT image analysis methods to extract grain traits aimed at the wheat grain [26–29]. In this work, the time consumption of CT scanning is set as 18 seconds, which leads to the spatial resolution of CT scanning for rice panicles (~300 *μ*m) is lower than the spatial resolution of CT scanning for wheat spikes (68.8 *μ*m), thus the existing image analysis methods [26–28] are difficult to be used for the rice panicle image processing in our work. In addition, there is more serious adhesion between rice grains, and the traits extracted from wheat grain are not the same as those of rice grain. Therefore, this paper proposes an image processing and analysis method which is suitable for rice panicle image processing and grain 3D traits extraction, and it has been verified that this method can be extended to the whole rice grain trait measurement, which will promote the research of rice panicle dynamic development and rice nondestructive yield estimation in future.

## 4. Conclusion

This study proposed a novel method for measuring twenty-two 3D rice grain traits using X-ray computed tomography. The *R*^2^ values between the CT and YTS measurements of the grain number and grain length were 0.980 and 0.960, respectively; that is, we found that the *R*^2^ value between the total grain volume and grain weight could reach as high as 98.0%. In short, compared with 2D imaging methods, the proposed method has several advantages; for example, no threshing is required, and the proposed technique can extract new 3D grain traits, such as 3D grain volume and grain density, reflected the grain size and grain quality, respectively. In addition, using the measured traits to build models effectively achieved the classification of rice varieties. In future, combined with genome-wide associate study or QTL analysis, the new 3D grain traits would provide more valuable information for the genetic architecture of rice grains, which will promote rice functional genomics and rice breeding.

## Figures and Tables

**Table 1:** Digitally extracted results of 22 traits per panicle by X-ray CT.

	Traits	Abbreviation
Grain number	Grain number	GN
Grain shape	Mean value of grain length	MGL
	Standard deviation of grain length	SGL
	Mean value of grain width	MGW
	Standard deviation of grain width	SGW
	Mean value of grain thickness	MGT
	Standard deviation of grain thickness	SGT
	Mean value of grain length/width ratio	MLWR
	Standard deviation of grain length/width ratio	SLWR
	Mean value of grain width/thickness ratio	MWTR
	Standard deviation of grain width/thickness ratio	SWTR
	Mean value of equivalent diameter	MED
	Mean value of solidity	MS
Grain size	Total grain volume	TGV
	Mean value of grain volume	MGV
	Standard deviation of grain volume	SGV
	Total grain surface area	TGS
	Mean value of grain surface area	MGS
	Standard deviation of grain surface area	SGS
	Mean value of convex hull volume	MCHV
	Standard deviation of convex hull volume	SCHV
Grain density	Mean value of grain grayscale value	MGG

**Table 2:** Comparison results of the different model for rice varieties classification (%).

Model	1	2	3	4	5	6	7	8	9	Average
SDA	95.8	100.0%	100.0%	90.0	80.0	100.0%	70.0	100.0%	90.0	92.3
SVM	95.8	100.0%	90.0	90.0	100.0%	90.0	90.0	100.0%	90.0	94.2
RF	95.8	100.0%	70.0	100.0%	100.0%	100.0%	90.0	80.0	90.0	92.3

## Supplementary Materials

Supplementary Materials Supplementary File 1: Original phenotypic data of all 104 rice panicles. Supplementary File 2: Usage instructions for the 3D image analysis pipeline. Supplementary File 3: Source code of the 3D image analysis pipeline. Supplementary Video 1: Guidelines of the operation procedure for the 3D image analysis pipeline. Supplementary Video 2: Example segmentation result of a single panicle. Supplementary Video 3: Segmentation results of all the panicles in one rice plant
