# Supplementary material for: Nondestructive 3D Image Analysis Pipeline to Extract Rice Grain Traits Using X-Ray Computed Tomography
Source: Plant Phenomics. 2020 May 2;2020:3414926. doi: 10.34133/2020/3414926 (PMC7706343; doi:10.34133/2020/3414926)
Supplement: Supplementary Materials — Supplementary File 1: Original phenotypic data of all 104 rice panicles. Supplementary File 2: Usage instructions for the 3D image analysis pipeline. Supplementary File 3: Source code of the 3D image analysis pipeline. Supplementary Video 1: Guidelines of the operation procedure for the 3D image analysis pipeline. Supplementary Video 2: Example segmentation result of a single panicle. Supplementary Video 3: Segmentation results of all the panicles in one rice plant [file 3414926.f1.zip › 3414926.f2.pdf]

Usage Instructions Of Software For Extraction Rice Grain Traits

Contents

1 Usage……………………………………………………………………..2

1.1 Input configuration……………………………………………………..2

1.2 Running…………………………………………………………………2

2 Files and Functions……………………………………………………......2

2.1 batchProcessing…………………………………………………………2

2.2 getFolderList……………………………………………………………2

2.3 readTif, saveTif…………………………………………………………2

2.4 volumeCreate……………………………………………………………2

2.5 createMask………………………………………………………………2

2.6 removeBlank……………………………………………………………3

2.7 calcGrainSize……………………………………………………………3

2.8 calcSurfaceArea…………………………………………………………3

2.9 traitsSummary…………………………………………………………..3

2.10 calcTotalTraits…………………………………………………………3

2.11 applySplit………………………………………………………………3

3 Output……………………………………………………………………...3

1 Usage

Usage of this software is very simple, and only a small amount of input is required, including the path to read the data and to save the result.

1.1 Input configuration

Configure the path to read data and to save the result and select whether to save intermediate processed image result. Set sampleNum value to specify the number of samples to process. samplesNum = -1 represents processing all samples.

```
% The root path of the data to be read
rootPath='D:\panicleData';
% The path to save the result
savePath='D:\Test\20191229ricepanicle';
% Whether to save the intermediate processed image results
isSaveImage=1;
% Specify the number of files to process
sampleNum=-1;
```

1.2 Running

Runing this program is as simple as calling batchProcessing function. Open the setup. M file and click Run.

```
% Will handle all folders under the root directory
batchProcessing(rootPath, savePath, sampleNum, isSaveImage);
```

2. Files and Functions

2.1 batchProcessing

batchProcessing is the main control function of this program, which is responsible for calling other functions to perform calculations and saving results.

2.2 getFolderList

getFolderList will Get all the folders under the root directory, and delete the non-conforming items, return the folder list.

2.3 readTif, saveTif

Read and save image files in .tif format.

2.4 volumeCreate

volumeCreate is used to read a series of slice image, by stacking all slice along the z direction, return 3D volume image.

2.5 createMask

createMask will return a mask to remove the holder in the image by detecting the inner edge of the holder.

2.6 removeBlank

removeBlank is used to remove blank areas around object, reduce image size and speed up calculations.

2.7 calcGrainSize

calcGrainSize calculates the length, width, thickness of individual grain.

2.8 calcSurfaceArea

calcSurfaceArea measures the surface area in voxels of a 3D object.

2.9 traitsSummary

traitsSummary brings together the traits of all samples.

2.10 calcTotalTraits

calcTotalTraits calculates all traits.

2.11 applySplit

applySplit splits connected objects.

3. Output

3.1 There are files under each folder under the output folder as follows:
a .csv file for saving single grain trait.
a .csv file for saving rice panicle trait.
a .tif file for saving the result of intermediate image processing.

grainbw.tif	2019/12/29 1:33	TIF 文件	2,041 KB
panicleTraits.csv	2019/12/29 1:33	Microsoft Excel ...	1 KB
singleGrainTraits.csv	2019/12/29 1:33	Microsoft Excel ...	7 KB

3.2 a statistics csv file of all sample traits.

totalTraits.csv	2019/11/25 13:05	Microsoft Excel ...	20 KB
